# TRPA1 and TRPV1 are required for lidocaine-evoked calcium influx and neuropeptide release but not cytotoxicity in mouse sensory neurons

**DOI:** 10.1371/journal.pone.0188008

**Published:** 2017-11-15

**Authors:** Mirjam Eberhardt, Thomas Stueber, Jeanne de la Roche, Christine Herzog, Andreas Leffler, Peter W. Reeh, Katrin Kistner

**Affiliations:** 1 Institute of Physiology and Pathophysiology, Friedrich-Alexander-University Erlangen-Nuernberg, Erlangen, Germany; 2 Department for Anaesthesia and Critical Care Medicine, Hannover Medical School, Hannover, Germany; 3 Institute of Neurophysiology, Hannover Medical School, Hannover, Germany; Indiana University School of Medicine, UNITED STATES

## Abstract

**Background:**

Local anaesthetics (LA) reduce neuronal excitability by inhibiting voltage-gated Na^+^ channels. When applied at high concentrations in the direct vicinity of nerves, LAs can also induce relevant irritation and neurotoxicity via mechanisms involving an increase of intracellular Ca^2+^. In the present study we explored the role of the Ca^2+^-permeable ion channels TRPA1 and TRPV1 for lidocaine-induced Ca^2+^-influx, neuropeptide release and neurotoxicity in mouse sensory neurons.

**Methods:**

Cultured dorsal root ganglion (DRG) neurons from wildtype and mutant mice lacking TRPV1, TRPA1 or both channels were explored by means of calcium imaging, whole-cell patch clamp recordings and trypan blue staining for cell death. Release of calcitonin gene-related peptide (CGRP) from isolated mouse peripheral nerves was determined with ELISA.

**Results:**

Lidocaine up to 10 mM induced a concentration-dependent reversible increase in intracellular Ca^2+^ in DRG neurons from wildtype and mutant mice lacking one of the two receptors, but not in neurons lacking both TRPA1 and TRPV1. 30 mM lidocaine also released Ca^2+^ from intracellular stores, presumably from the endoplasmic reticulum. While 10 mM lidocaine evoked an axonal CGRP release requiring expression of either TRPA1 or TRPV1, CGRP release induced by 30 mM lidocaine again mobilized internal Ca^2+^ stores. Lidocaine-evoked cell death required neither TRPV1 nor TRPA1.

**Summary:**

Depending on the concentration, lidocaine employs TRPV1, TRPA1 and intracellular Ca^2+^ stores to induce a Ca^2+^-dependent release of the neuropeptide CGRP. Lidocaine-evoked cell death does not seem to require Ca^2+^ influx through TRPV1 or TRPV1.

## Introduction

Local anesthetics (LA) like lidocaine reduce the excitability of neurons to prevent or relieve the perception of pain primarily by reversibly inhibiting voltage-gated sodium channels [1]. LAs are very commonly used for diverse forms of local, regional or spinal anesthesia, and their use is associated with a rather low prevalence of severe side effects. Besides systemic cardiac and central nervous side effects, however, the local administration of LAs at high concentrations may lead to inadvertent effects such as pain upon injection, local tissue damage, inflammation and direct neurotoxicity [2]. Relevant neurotoxic effects seem to occur predominantly after intrathecal application, are concentration-dependent and can range from reversible pain or dysesthesia associated with transient neurological symptoms to irreversible nerve conduction block causing a cauda equina syndrome [3–7]. According to the substantial clinical relevance associated with severe LA-induced neurotoxic effects, numerous *in vitro* studies were performed in order to unravel mechanisms mediating LA-induced neurotoxicity. Several molecular mechanisms have meanwhile been suggested to contribute to LA-induced necrosis or apoptosis observed in different cellular systems. In a recent overview, Verlinde and co-workers presented intracellular signaling pathways involving caspase, phosphoinositide 3-kinase and mitogen-activated protein kinase as proposed key mechanisms for LA-induced cytotoxicity [7]. From our point of view, a relevant question should also be how LAs activate these pathways, e.g. which upstream mechanisms are employed by LAs to activate certain intracellular pathways leading to cell death? However, relatively little seems to be known about such mechanisms to date. While inhibition of voltage-gated sodium channels per se does not seem to be neurotoxic [6], some studies found that LA-induced cytotoxicity is associated with an increase in intracellular calcium [8–10]. In fact, removal or buffering of extracellular calcium strongly attenuated lidocaine-induced cell death in rat sensory neurons [9], suggesting that calcium influx through the cell membrane might trigger LA-induced cell damage.

Our laboratories previously demonstrated that clinically applied concentrations of lidocaine and other LAs directly activate the ion channels TRPV1 and TRPA1 [11,12]. Both channels are predominantly expressed in nociceptive afferent C-fibers, are calcium-permeable and can be activated by a large variety of irritant and painful stimuli [13]. Besides being important transduction molecules in nociceptors, activation of both TRPA1 and TRPV1 results in a calcium-dependent neuropeptide release. This could initiate further events like nociceptor sensitization, inflammation and vasodilation (neurogenic inflammation) [13,14]. While we already demonstrated that TRPV1 is relevant for lidocaine-evoked calcium influx and release of neuropeptides in mouse sensory neurons [11], the relevance of TRPA1 as a target for LAs in sensory neurons is largely unknown. Both TRPV1 and TRPA1 can mediate cytotoxicity upon activation [15–19], and calcium influx seems to be required at least for cytotoxicity mediated by TRPV1 [20]. Thus, it is possible that TRPV1 and TRPA1 are important calcium shuttles involved in lidocaine-evoked toxicity in sensory neurons.

In the present study we employed sensory neurons as well as intact nerves from wildtype and different mutant mice in order to describe the role of TRPA1 and TRPV1 for lidocaine-evoked calcium-influx, membrane depolarization, neuropeptide release and neurotoxicity in detail.

## Materials and methods

### Animals

All animal procedures were approved by the animal protection authority of the local district government (Regierung von Mittelfranken, Ansbach, and Gewerbeaufsicht, Niedersachsen, Germany). Adult mice (8–12 weeks, 18–25 g) of both sexes were housed in groups under temperature- and air humidity-controlled conditions in a 12-h light-dark cycle and had access to food and water *ad libitum*. For all experiments animals were sacrificed in a rising carbon dioxide atmosphere. TRPA1-knockout (TRPA1^-/-^) mice were donated by D. P. Corey (Harvard University, USA), TRPV1-knockout mice were a gift from J. B. Davis (formerly Glaxo-Smith-Kline, UK). TRPV1/TRPA1-double knockout (TRPV1/TRPA1 ^= / =^) mice were inbred in our animal facility and have become congenic by backcrossing to native C57BL/6 mice for at least six generations. Each knockout mouse was conventionally genotyped before employing it in experimental procedures.

### Cell culture

Dorsal root ganglia (DRG) from C57BL/6, TRPV1^-/-^, TRPA1^-/-^, and TRPV1/TRPA1 ^= / =^ mice were removed and transferred into Dulbecco’s modified Eagle’s medium solution (DMEM, Invitrogen) containing 50 μg/ml gentamicin (Sigma-Aldrich). Ganglia were digested in 1 mg/ml collagenase and 0.1 mg/ml protease (both Sigma-Aldrich) for 30 min. After centrifugation the cell suspension was dissociated using a fire-polished silicone-coated Pasteur pipette. Cells were plated onto poly-D-lysine-coated (200 μg/ml; Sigma-Aldrich) 12-mm glass tissue culture dishes with/without 12-well-plates (Greiner Bio-One) and cultured in TNB 100 cell culture medium supplemented with TNB 100 lipid-protein complex (Biochrom), 100 μg/ml streptomycin, penicillin (PAA Laboratories), and 100 ng/ml mouse NGF (Alomone Labs) at 37°C in 5% carbon dioxide. Experiments were performed within 12–24 h of dissociation.

### Ratiometric [Ca^2+]^_i_ measurements

DRG neurons of TRPV1^-/-^ and TRPV1^-/-^ TRPA1^-/-^ mice were cultured on Poly-D-Lysin coated coverslips. Cells were stained by 5 μM Fura-2 AM in 0.02% Pluronic (both from Invitrogen) dissolved in TNB medium for about 30 min. Following a 30 min wash out period to allow Fura-2 AM deesterification, coverslips were mounted on an Olympus IX71 inverse microscope with a 20x objective. DRG neurons were constantly superfused with extracellular fluid (in mM: NaCl 145, KCl 5, CaCl_2_ 1.25, MgCl_2_ 1, glucose 10, HEPES 10) using a software controlled 7-channel gravity driven common-outlet superfusion system. Fura-2 was excited at 340 and 380 nm with a Polychrome V monochromator (Till Photonics). Images were exposed for 200 μs and acquired at a rate of 1 Hz with a 12–bit CCD camera (Imago Sensicam QE, Till Photonics). Data were recorded and further analyzed using TILLvisION 4.0.1.3 software (Till Photonics). Background fluorescence was subtracted before calculation of ratios. Calculation of [Ca^2+^]_i_ was performed, and increases by more than 50 nM were considered positive. For depletion of intracellular calcium stores thapsigargin 2 μM was added for 20 minutes after Fura-2 AM staining of the neurons followed by a 10 min wash out to allow 30 min of Fura-2 deesterification as in untreated neurons. This treatment proved sufficient to abolish the response of the neurons to a 2 mM caffeine stimulus targeting intracellular calcium stores (data not shown). Summarized results are reported as area under the curve in percent of increase evoked by a 60 mM potassium chloride stimulus which was used as a control at the end of each experiment. NaCl concentration was reduced in these solutions to maintain constant osmolarity.

### Patch-clamp electrophysiology

Whole-cell voltage-clamp recordings were performed on cultured DRG neurons of C57BL/6, TRPV1^-/-^, TRPA1^-/-^ and TRPV1/TRPA1 ^= / =^ mice. The standard extracellular solution contained the following in mM: 140 NaCl, 5 KCl, 2 CaCl_2_, 2 MgCl_2_, 10 HEPES, and 10 glucose at pH 7.4 (adjusted by Tetramethylammonium hydroxide). Pipette solution contained in mM: 140 KCl, 2 MgCl_2_, 5 EGTA, and 10 HEPES (pH = 7.4 by KOH). Solutions were bath applied using a gravity-driven polytetrafluoroethylene multibarrel perfusion system (custom-made). All recordings were performed at room temperature. Transmembrane ion currents were acquired using an Axopatch 200B amplifier (Molecular Devices). Data were low-pass filtered at 2 kHz and sampled at 5 kHz. Patch pipettes were pulled from thick-walled borosilicate glass capillaries (GB150F-8P, Science Products) and heat-polished to give a resistance of 1.5–3.0 MΩ. Unless otherwise noted, cells were held at a potential of -60 mV. pCLAMP 10.3 (Molecular Devices) was used for data acquisition, digitization and for further offline analysis.

### Release of calcitonin gene-related peptide (CGRP)

Sciatic nerves of C57BL/6, TRPV1^-/-^, TRPA1^-/-^ and TRPV1/TRPA1 ^= / =^ mice were exposed and excised from their emergence out of the lumbar plexus to their branching into tibial, sural, and peroneal nerves. Preparations were loosely wrapped around acrylic rods, placed in carbogen-gassed (95% O_2_ and 5% CO_2_) synthetic interstitial fluid (SIF) containing (in mM) 108 NaCl, 3.48 KCl, 3.5 MgSO_4_, 26 NaHCO_3_, 1.7 NaH_2_PO_4_, 1.5 CaCl_2_, 9.6 sodium gluconate, 5.5 glucose, and 7.6 sucrose, and positioned in a thermostatic shaking bath at 32°C. After an initial wash-out period of 30 min all subsequent experiments were performed in glass reaction tubes at 32°C and each individual incubation step lasted for 5 minutes. By the first two incubations basal CGRP release was determined in reaction tubes containing SIF solution, the third incubation assessed the response to chemical stimulation and test tubes contained lidocaine 10 or 30 mM. The final incubation period in SIF allowed for recovery of the stimulated CGRP release. Antagonists used in a set of experiments were added to SIF in the second step and in combination with lidocaine during the third incubation period. CGRP content of incubation fluids was measured using a commercial enzyme immunoassay kit (Bertin Pharma) with a detection limit of 5 pg/ml. Samples were analyzed photometrically using a 96-well microplate reader (Dynatech). For graphs presenting the stimulated CGRP release data (in pg/ml), baseline levels from the second incubation period were subtracted from all CGRP values obtained.

### Cell viability

DRG neurons from wild type (n = 6) and TRPV1/TRPA1-double knockout (n = 4) mice were cultured as described under cell culture. Dissociated DRG neurons were cultivated on glass coverslips in 12-well plates allowing pharmacological experiments on separate coverslips. The experimental protocol for examining lidocaine-evoked cytotoxicity on DRG neurons was derived from a previous study [9]. Lidocaine at 10 and 30 mM was added to the medium for 15 min, followed by washout and replacement with fresh medium. After 1 h or 24 h incubation, cells were stained with trypan blue (0.04%, Sigma-Aldrich) for 10 min. Phase contrast photographs (20x) were taken from four representative visual fields of each coverslip with a CCD camera (Cool SNAP EZ Photometrics) installed on an inverse microscope (Axio Observer D1, Zeiss). Cytotoxicity was quantified by manual counting of the percentage of cells stained with trypan blue relative to the total number of DRG neurons. The experimenter was blinded to the experiments.

Apoptosis was detected and measured using the Cell-APOPercentage apoptosis assay (Biocolor, Tebu-bio) which uses a dye that is selectively imported by cells that are undergoing apoptosis. After staining the cells four pictures per well were taken using cell∧F imaging software (Olympus IX81, Olympus Europa Holding GmbH). The total number of cells and the number of apoptotic cells were counted using ImageJ 10.2 analysis software (NIH).

Stable hTRPV1-expressing HEK293 cells (a kind gift from Peter Zygmunt, Lund, Sweden), hTRPA1-transfected HEK 293 cells and native HEK 293t cells were cultured in DMEM (D-MEM, Gibco) supplemented with 10% FBS (Biochrom), 100 U/ml penicillin and 100 μg/ml streptomycin and 2 mM Glutamax (all Gibco). 5 μg/ml blasticidin (PAA Laboratories) and 0.35% Zeocin (Invitrogen) were added for stable expression of hTRPV1 and for induction tetracycline 0.1 μg/ml (Sigma-Aldrich) was added to the medium 16 to 24 h prior to experiments. A stable hTRPA1 HEK293 cell line was established using G418 800 μg/ml (Sigma-Aldrich) as described previously [21]. Cells were cultivated at 37°C and 5% CO_2_ and prepared in 12-well plates (Greiner Bio-One). 10 or 30 mM lidocaine was added to the cell medium for 1 h, followed by washout and replacement with medium. After 1 h incubation cells were resuspended and stained with trypan blue (0.04%, Sigma-Aldrich) for 10 min. Staining with trypan blue was evaluated by automatic counting in a Cellometer (Nexcelom Bioscience).

### Drugs

Chemical solutions were prepared by dilution of frozen aliquots of stock solutions in extracellular solution on the day of each experiment.

Lidocaine (2-diethylamino-*N*-(2,6-dimethylphenyl)acetamide) as hydrochloride was dissolved directly in extracellular solution. For capsaicin (8-methyl-N-vanillyl-trans-6-nonenamide), BCTC (4-(3-chloro-2-pyridinyl)-N-[4-(1,1-dimethylethyl)phenyl]-1-piperazinecarboxamide,), HC-030031 (1,2,3,6-tetrahydro-1,3-dimethyl-N-[4-(1-methylethyl)phenyl]-2,6-dioxo-7H-purine-7-acetamide) stock solutions were prepared in absolute ethanol, while for allyl isothiocyanate (AITC) and thapsigargin, and dimethyl sulfoxide was used (all compounds from Sigma-Aldrich). If necessary, pH values of test solutions were adjusted to pH 7.4 with NaOH or HCl, respectively, before use.

### Statistical analysis

All Data are presented as mean ± S.E.M. Sample sizes for all experiments were chosen in accordance to standard practice in the field and are defined in figure legends. Data were analyzed using Wilcoxon matched pairs test for intraindividual, Mann–Whitney *U* test and unpaired t-test, respectively, for group comparisons or one way ANOVA followed by HSD post hoc tests for comparison of more than two groups. Respective statistical tests used are denoted in the text and/or figure legends. Differences were considered statistically significant at *p* values *<* 0.05 and marked with *, *p* values < 0.01 are indicated as **, *p* values < 0.001 as ***. Calculations for statistical analysis were performed with the Statistica software package 7.1 (StatSoft).

## Results

### Lidocaine-induced calcium influx in DRG neurons depends on TRPA1 and TRPV1

**Fig 1:**
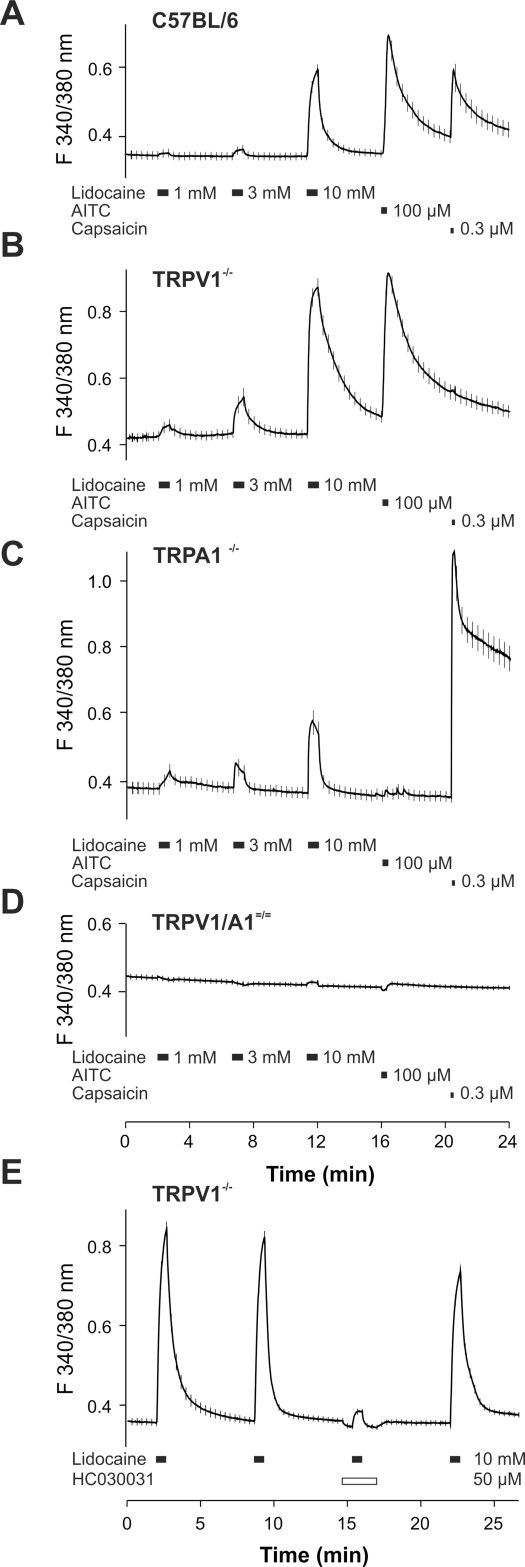
Lidocaine-induced increase in [Ca^2+^]i involves TRPA1 and TRPV1 receptors in DRG neurons. **A- D.** DRG neurons of C57Bl/6, TRPV1^-/-^, TRPA1^-/-^ and TRPV1/A1^-/-^ mice were exposed to increasing concentrations (1- 10mM) of lidocaine for 40 s (indicated by bars). Traces show averaged responses of lidocaine-responsive cells (A-E), or all measured neurons in TRPV1/A1^-/-^ mice and are presented as mean +/- S.E.M. The S.E.M is depicted as a small vertical line along the drawn line depicting the average response (n≥ 31 for each group). **E.** The TRPA1 inhibitor HC030031 (50 μM) completely blocks the lidocaine-induced [Ca^2+^]_i_ increase in TRPV1^-/-^ DRG neurons (p = 0.000008, ANOVA F(3, 336) = 110.98, p = 0.0000, followed by HSD post hoc test).

In a previous study we have already demonstrated that TRPV1 substantially contributes to calcium influx evoked by 30 mM lidocaine in mouse and rat DRG neurons [11]. Therefore, we first examined the role of TRPA1 for lidocaine-induced intracellular calcium ([Ca^2+^]_i_) increase by conducting ratiometric calcium measurements on DRG neurons from adult mice. Neurons were exposed to increasing concentrations of lidocaine (1, 3, 10 mM) for 40 s at intervals of 4 minutes. The TRPA1 agonist allyl isothiocyanate (AITC, 100 μM), the TRPV1 agonist capsaicin (0.3 μM) and potassium chloride (KCl, 60 mM, data not shown) were used as control stimuli for functional expression of TRP channels and for cell viability, respectively. Lidocaine produced a concentration-dependent increase in [Ca^2+^]_i_ in neurons from wild type C57BL/6 (Fig 1A), TRPV1^-/-^ (Fig 1B) and TRPA1^-/-^ knockout (Fig 1C) mice. In neurons from TRPV1/TRPA1 double knockout mice, however, lidocaine up to 10 mM almost completely failed to raise [Ca^2+^]_i_ (Fig 1D). While 64% of all examined DRG neurons from wild type mice responded to 10 mM lidocaine, the corresponding percentages were reduced to 53% in neurons lacking TRPV1, to 17% in TRPA1^-/-^ neurons and to only 4% of neurons from TRPV1/TRPA1 ^= / =^ mice. In order to further substantiate the role of TRPA1 as a sensor for lidocaine in DRG neurons, calcium influx was repeatedly induced by application of 10 mM lidocaine to DRG neurons lacking TRPV1. This approach resulted in robust and reproducible calcium transients (Fig 1E). When lidocaine was applied together with the TRPA1 inhibitor HC030031 (50 μM), no calcium influx was elicited compared to two foregoing and one subsequent application of lidocaine (p<0.000008 compared to each lidocaine application without HC030031; Fig 1E; all ANOVA followed by HSD post hoc test).

**Fig 2:**
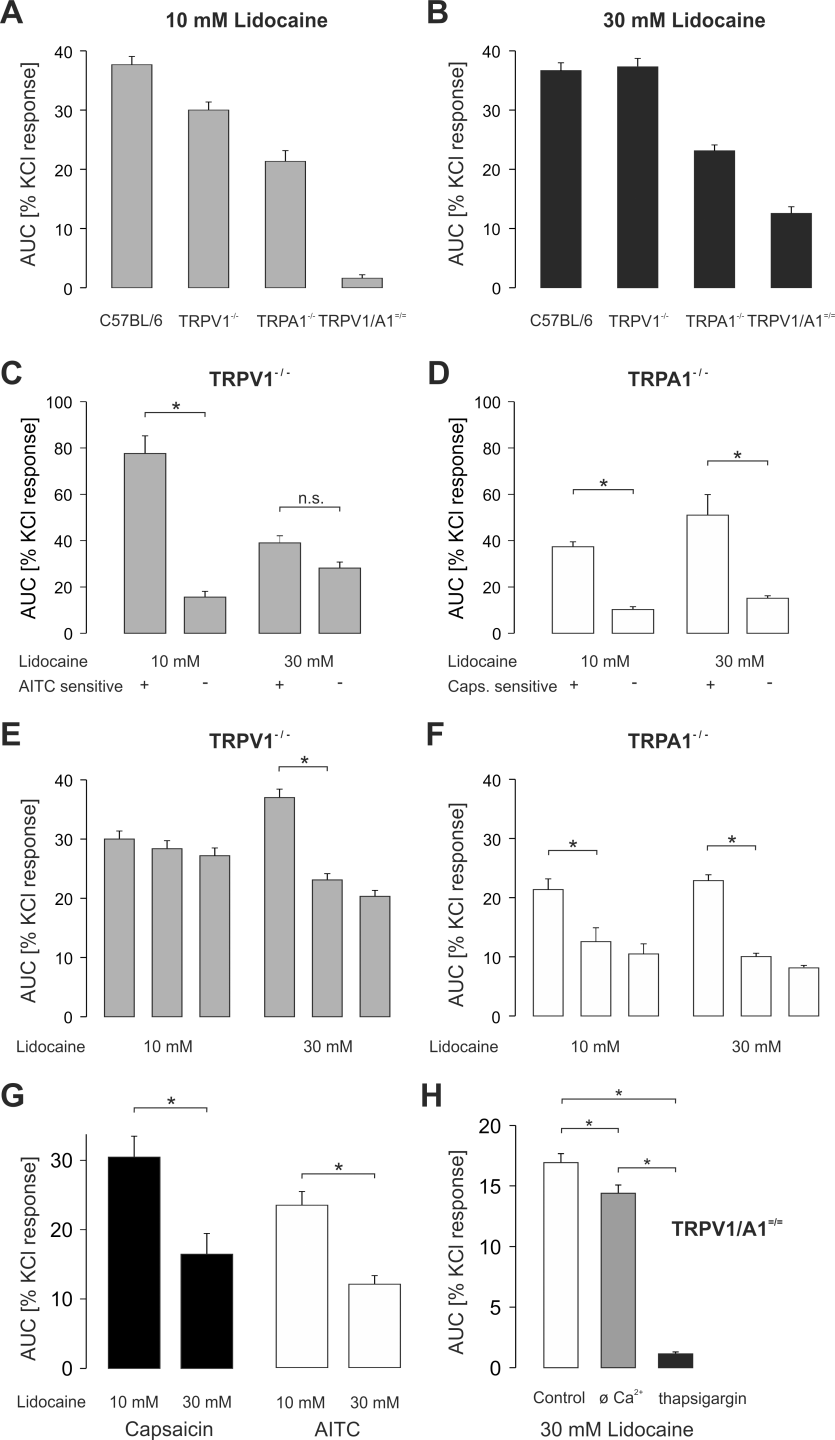
Areas under the curve (AUC) of increases in [Ca^2+^]_i_ evoked by lidocaine. **A-B.** Areas under the curve of increases in intracellular calcium evoked by lidocaine 10 mM (A) and 30 mM (B) in DRG neurons of C57BL/6, TRPV1^-/-^, TRPA1^-/-^ and TRPV1/A1^-/-^ mice. Calcium responses to lidocaine stimuli significantly differ among the different genotypes (p ≤ 0.001), while at 30 mM lidocaine-evoked increase in [Ca^2+^]_i_ is similar in neurons of C57BL/6 and TRPV1^-/-^ mice (p = 1.0). **C-D.** Calcium responses to 10 and 30 mM lidocaine compared between AITC-positive and AITC-negative DRG neurons from TRPV1^-/-^ mice (C) or between capsaicin-positive and capsaicin-negative DRG neurons from TRPA1^-/-^ mice (D). * indicates p≤ 0.016 each. **E-F.** Calcium responses to three subsequent lidocaine stimuli show prominent desensitization, which involves TRPA1 receptors at 30 mM lidocaine (E; p = 0.00002) and TRPV1 receptors at 10 and 30 mM (F; p = 0.0002 and p = 0.00002). **G.** Increases in intracellular calcium evoked by capsaicin (0.3μM, black) or AITC (100μM, white bars) desensitize to a larger extent following 30 mM than after 10 mM lidocaine (p≤ 0.003; each). **H**. Lidocaine evokes calcium responses even in DRG neurons of TRPV1/A1^-/-^ mice, which were reduced in calcium free extracellular solution (p = 0.004), but nearly abolished only by depletion of intracellular calcium stores by pretreatment with thapsigargin (2 μM, p = 0.00002). Data are normalized to a final 60 mM potassium stimulus applied to the neuros to evoke maximal calcium influx. Error bars represent S.E.M. (all ANOVA followed by HSD post hoc tests calculated for each panel separately apart from data presented in 2A+B).

Taken together, these data strongly suggest that TRPA1 significantly contributes to lidocaine-evoked calcium influx at concentrations up to 10 mM. Furthermore, they also support our previous findings demonstrating that TRPV1 participates as a sensor for lidocaine as well. In order to further dissect the individual contributions of TRPA1 and TRPV1 as receptors activated by lidocaine in DRG neurons, we next compared the area under the curve (AUC) of [Ca^2+^]_i_ following application of 10 and 30 mM lidocaine in neurons from wild type, TRPV1^-/-^, TRPA1^-/-^ and TRPV1/TRPA1 ^= / =^ mice. Intracellular calcium following lidocaine had returned to baseline before AITC and capsaicin were applied as controls to exclude superimposition of responses. Compared to neurons responding to 10 mM lidocaine in wild type mice, the AUC of this effect was significantly reduced in neurons from both TRPV1^-/-^ and TRPA1^-/-^ mice (Fig 2A; p≤0.0004). While the absence of TRPA1 resulted in a significantly greater reduction of the sensitivity to 10 mM lidocaine compared to the TRPV1 deficit (p = 0.004), neurons from mice lacking both receptors generated only a minimal increase in [Ca^2+^]_i_ when challenged with 10 mM lidocaine (Fig 2A). When lidocaine was applied at 30 mM, however, neurons from wild type and TRPV1^-/-^ mice displayed similar responses (Fig 2B; p = 1.0). In contrast, neurons lacking TRPA1 displayed significantly reduced effects compared to neurons of wild type and TRPV1^-/-^ mice (Fig 2B; p = 0.00003 each). As expected, neurons from TRPV1/TRPA1 ^= / =^ mice produced the smallest responses to 30 mM lidocaine (Fig 2B; p≤0.00005). However, it was evident that 30 mM lidocaine evoked a greater increase in intracellular calcium in double knockout neurons than 10 mM lidocaine (p = 0.00003). Moreover, the percentage of double knockout neurons responding to lidocaine increased from 4% at 10 mM to 42% at 30 mM lidocaine. These data indicate that activation of TRPA1 is the main mechanism for lidocaine-evoked increase in [Ca^2+^]_i_ in DRG neurons, and that TRPV1 seems to play only a minor role. In order to further validate this interpretation, we next separately calculated the AUCs of lidocaine-evoked increase in [Ca^2+^]_i_ in TRPA1-expressing (i.e. responsive to AITC) neurons versus neurons lacking TRPA1 (i.e. insensitive to AITC) from TRPV1^-/-^ mice. As demonstrated in Fig 2C, expression of TRPA1 was associated with a significantly greater response to 10 mM lidocaine (p = 0.00008). However, this difference was lost at 30 mM lidocaine (p = 0.16), indicating that at higher concentrations lidocaine-induced increase in intracellular calcium is also mediated by mechanisms other than TRPA1 activation. The same approach was performed for TRPV1, i.e. in neurons of TRPA1^-/-^ mice the AUCs of [Ca^2+^]_i_ were separately calculated for those expressing TRPV1 (i.e. sensitive to capsaicin) and those not expressing TRPV1 (i.e. insensitive to capsaicin). While presence of TRPV1 resulted in a greater response to 10 mM lidocaine (p = 0.016), the response to 30 mM lidocaine was even larger in neurons expressing TRPV1 (p = 0.00003; Fig 2D).

We have previously demonstrated that lidocaine-evoked activation of recombinant TRPV1, and to a lesser extent of TRPA1, display a prominent desensitization [11,12]. Upon repeated application of 10 mM lidocaine, the responses in DRG neurons of mice lacking TRPV1 did not undergo strong desensitization but remained stable (p = 0.95; Fig 2E). With 30 mM lidocaine, however, we observed a strong desensitization following three consecutive applications of lidocaine (p = 0.00002; Fig 2E). In neurons lacking TRPA1, however, the responses resulting from application of both 10 and 30 mM lidocaine revealed a prominent desensitization (p≤0.0002; Fig 2F). These data indicate that lidocaine-evoked activation of TRPV1 and TRPA1 undergoes a concentration-dependent desensitization, and that this effect is stronger on TRPV1 than on TRPA1. We also explored if lidocaine induces a cross-desensitization towards activation by other agonists. For this purpose, application of capsaicin (0.3 μM, for activation of TRPV1) or AITC (100 μM, for activation of TRPA1) was preceded with the application of 10 or 30 mM lidocaine in C57BL/6 DRG neurons. Indeed, both capsaicin- and AITC-induced effects were significantly smaller following application of 30 mM lidocaine compared to 10 mM lidocaine (p≤0.003; Fig 2G). It is evident that 30 mM lidocaine also employs a mechanism independent of TRPA1 and TRPV1 to increase [Ca^2+^]_i_. Knowing very little about the mechanism/s that could mediate this effect, we next asked if the increase in [Ca^2+^]_i_ evoked by 30 mM lidocaine involves extracellular and/or intracellular calcium. When 30 mM lidocaine was applied in calcium free external solution, responses in double knockout DRG neurons were only slightly smaller compared to the effects observed in the presence of extracellular calcium (p = 0.004; Fig 2H). Furthermore, depletion of intracellular calcium stores achieved by pre-treatment of DRG neurons with 2 μM thapsigargin for 20 minutes effectively abolished retained calcium responses to 30 mM lidocaine in TRPV1/TRPA1 ^= / =^ DRG neurons (p = 0.00002; Fig 2H; all ANOVA followed by HSD post hoc test). Thus, high concentrations of lidocaine seem to evoke a release of calcium from internal stores.

### Lidocaine-evoked inward currents in DRG neurons are mediated by TRPA1 and TRPV1

**Fig 3:**
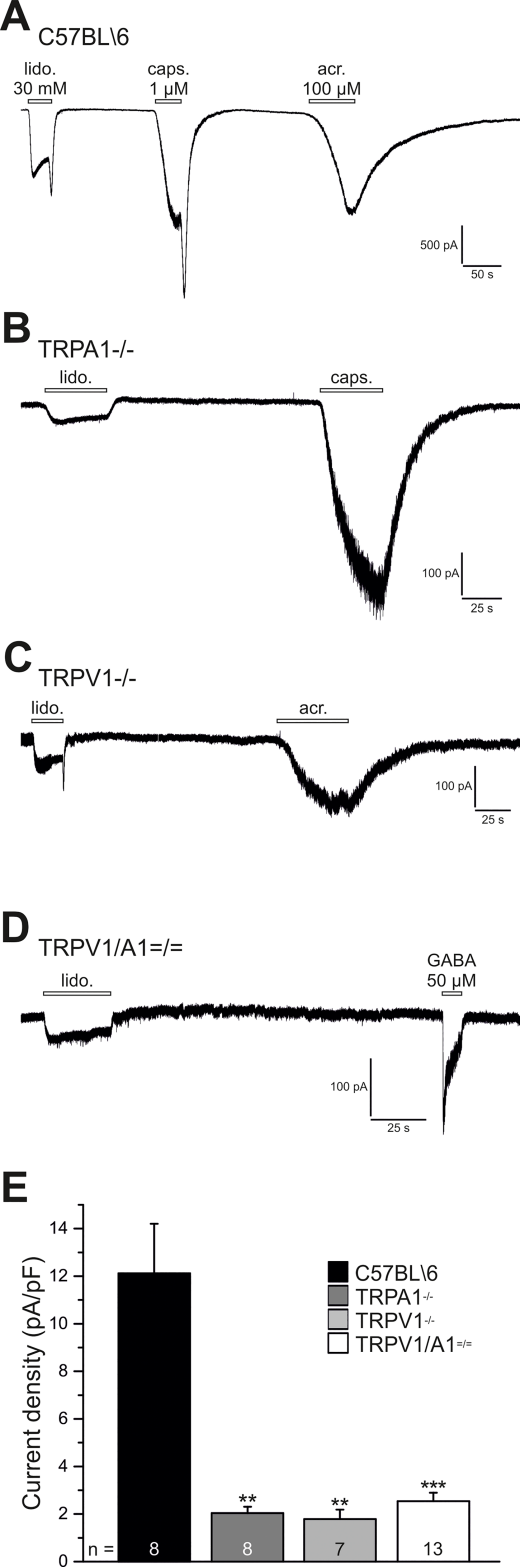
Lidocaine-activated inward currents are mediated by TRPA1 and TRPV1. Representative current traces of whole-cell voltage-clamp recordings from DRG neurons using a holding voltage of– 60 mV. Application of lidocaine 30 mM for 30 s produced inward currents in cells from **A**. C57Bl/6, **B**. TRPA1^-/-^, **C**. TRPV1^-/-^, and **D**. TRPV1/TRPA1 ^= / =^ mice. Subsequent application at intervals of 2–3 min of capsaicin (1 μM, 10 s) and/or acrolein (100 μM, 30 s) verified expression of TRPV1 and/or TRPA1 in lidocaine positive neurons. Gamma-aminobutyric acid (GABA, 50 μM, 10 s) was applied comfirming viability of TRPV1/TRPA1 ^= / =^ ganglion neurons tested. **E.** Comprehensive recordings of mean peak currents evoked by lidocaine (30 mM) in C57Bl/6 (black bar), TRPA1^-/-^ (dark grey bar), TRPV1^-/-^ (light grey bar), and TRPV1/TRPA1 ^= / =^ (white bar) DRG neurons. The largest lidocaine-activated currents were observed in C57Bl/6 cells (n = 8), while current densities were significantly reduced in all three knockout mice strains to about the same level (TRPA1^-/-^: p = 0.001, n = 8, TRPV1^-/-^: p = 0.002, n = 7, TRPV1/TRPA1 ^= / =^: p = 0.0002, n = 13). Results are mean and SEM of mean peak currents by lidocaine. For statistical analysis Mann-Whitney *U* test was calculated.

Knowing that 30 mM lidocaine evokes an increase in [Ca^2+^]_i_ only partly by activating TRPA1, we next asked if lidocaine-evoked inward cation currents involve other mechanisms than TRPA1 and TRPV1 as well. Whole-cell voltage-clamp recordings were performed on DRG neurons derived from the same mutant mice as used for calcium measurements. In initial experiments, we observed that brief application of 10 mM lidocaine did not produce reproducible inward currents in DRG neurons (data not shown). Thus, 30 mM lidocaine was applied for 30 s on small to medium sized DRG neurons (capacitance: 24.3 ± 1.6 pF, n = 72) and functional expression of TRPA1 and TRPV1 was examined by a subsequent application of acrolein (100 μM, 30s) or capsaicin (1 μM, 10s). In all examined wild type neurons, lidocaine evoked small inward currents with a mean peak current density of 12.1 ± 2.1 pA/pF (Fig 3A and 3E, n = 8). Only 8 of 19 examined neurons from TRPA1^-/-^ mice produced lidocaine-evoked currents, and these were significantly smaller than the currents produced in wild type cells (2.0 ± 0.3 pA/pF, p = 0.001, Fig 3B and 3E). When applied to DRG neurons of TRPV1^-/-^ mice, 30 mM lidocaine elicited inward currents in 7 of 11 examined cells. The current density of these currents was also reduced as compared to wild type neurons (1.8 ± 0.4 pA/pF, p = 0.002, Fig 3C and 3E). In mice lacking both TRPA1 and TRPV1, 30 mM lidocaine evoked inward currents in a substantial fraction of neurons (13/29). The current density was 2.5 ± 0.4 pA/pF and again significantly smaller than in wild type neurons (p = 0.0002, all Mann-Whitney U Test, Fig 3D and 3E). When lidocaine was applied together with the unselective TRP channel pore blocker ruthenium red (10 μM), lidocaine-induced inward currents in double knockout neurons were completely blocked (n = 3, data not shown). Viability of TRPV1/TRPA1 ^= / =^ DRG neurons was further verified by subsequent application of the neurotransmitter gamma-aminobutyric acid (GABA, 50 μM, 10s). Extra-synaptic GABA_A_ receptors are known to be functionally expressed in a large majority of DRG neurons. Among all neurons tested, 73% were sensitive to GABA, while 45% were responsive to lidocaine. Responsiveness to lidocaine as well as GABA was found in only 9% of all investigated cells (Fig 3D).

### Lidocaine stimulates CGRP release through TRPA1 and TRPV1 in peripheral axons

**Fig 4:**
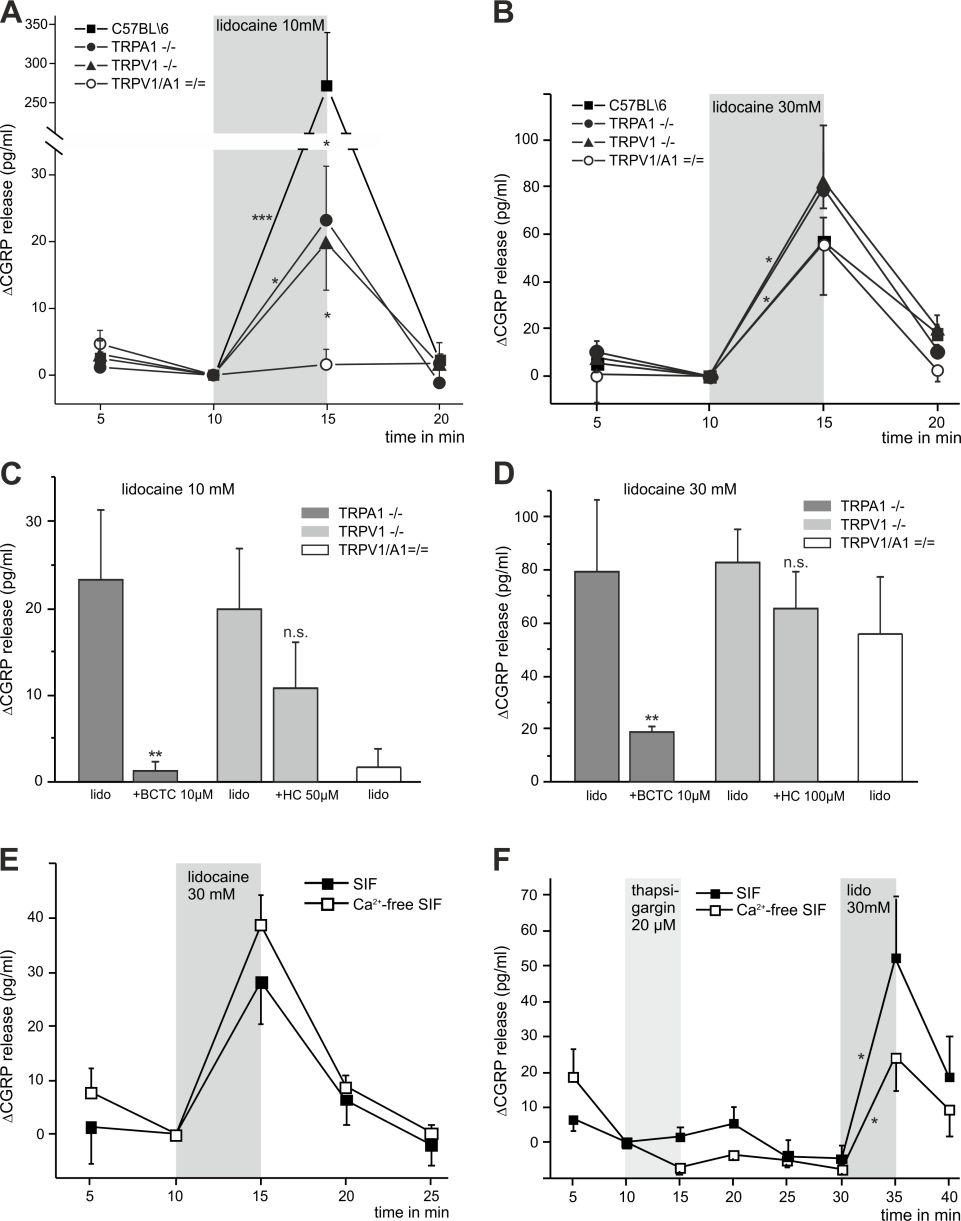
TRPA1 and TRPV1 differentially contribute to lidocaine-stimulated CGRP release from peripheral axons. Sciatic nerves from mice were isolated, stimulated with lidocaine and/or chemical substances (where stated) for 5 min, and induced CGRP-release over baseline was measured quantitatively. **A.** Lidocaine 10 mM and **B.** 30 mM stimulated CGRP release from C57BL/6 nerves (10mM: p = 0.0004, n = 16; 30 mM: p = 0.012, n = 8). In nerves from TRPA1^-/-^ and TRPV1^-/-^ mice less CGRP was released by lidocaine 10 mM (both p = 0.012, n = 8), while stimulation was without any effect on CGRP release from TRPV1/TRPA1 ^= / =^ nerves (p = 0.674, n = 8). **B.** Stimulation with 30 mM lidocaine induced release of CGRP of comparatively the same extent in nerves from TRPA1^-/-^, TRPV1^-/-^ (both p = 0.012) and TRPV1/TRPA1 ^= / =^ mice (p = 0.017, all n = 8). **C, D.** Effects of additional application of either the TRPV1 inhibitor BCTC (10 μM) in TRPA1^-/-^ (lidocaine 10 mM: p = 0.003, 30 mM: p = 0.002, both n = 8) or the TRPA1 blocker HC030031 (50 μM) in TRPV1^-/-^ (n.s., n = 8 each) **E.** In C57BL/6 nerves CGRP release stimulated by lidocaine (30 mM) was nearly the same in calcium free as in calcium containing solution (n = 4). **F.** Initial application of thapsigargin (20 μM, 5 min) reduced subsequent CGRP release evoked by lidocaine (30 mM) to the half in Ca^2+^-free extracellular solution (p = 0.028, n = 8). Data are presented as mean ± SEM. For intra-individual comparison the Wilcoxon matched pairs test and for group comparison the Mann-Whitney *U* test were calculated.

Measurement of stimulated release of the neuropeptide calcitonin gene-related peptide (CGRP) from primary afferent neurons can serve as a surrogate model of neurogenic inflammation reflecting irritation and pain [14]. We have previously demonstrated that TRPV1 is mediating lidocaine-induced release of CGRP [11]. Here we asked if TRPA1 and possibly further mechanisms contribute to lidocaine-stimulated CGRP release as well. Isolated sciatic nerves of mice were treated with lidocaine for 5 minutes and CGRP release was measured by ELISA. Stimulation with 10 mM lidocaine induced a massive CGRP release from nerves of wild type C57BL/6 mice (p = 0.0004, Fig 4A). In nerves of TRPA1^-/-^ mice 10 mM lidocaine-evoked release of CGRP was reduced by 92% compared to wild type nerves (p = 0.012, Fig 4A). A similar result was found in nerves lacking TRPV1 in which the release of CGRP was reduced by 93% when challenged by 10 mM lidocaine (p = 0.012, Fig 4A). In nerves derived from mice lacking both TRPA1 and TRPV1, 10 mM lidocaine completely failed to increase release of CGRP (p = 0.674). In addition, the TRPV1 inhibitor BCTC (10 μM) effectively inhibited lidocaine-evoked CGRP release from TRPA1^-/-^ nerves (p = 0.003, Fig 4C). The other way round, the TRPA1 blocker HC030031 (50 μM) did not significantly reduce the effects of 10 mM lidocaine in TRPV1^-/-^ nerves (p = 0.327, Fig 4C).

Surprisingly, 30 mM lidocaine was less effective than 10 mM lidocaine in releasing CGRP from stimulated wild type nerves (p = 0.012, Fig 4B). Furthermore, CGRP release by 30 mM lidocaine did not significantly differ between wild type, TRPA1^-/-^, TRPV1^-/-^ and even TRPV1/TRPA1 ^= / =^ mice (TRPA1^-/-^: p = 0.674, TRPV1^-/-^: p = 0.115, TRPV1/TRPA1 ^= / =^: p = 0.401, Fig 4B). However, CGRP release evoked by application of 30 mM lidocaine was significantly greater compared to 10 mM lidocaine in nerves from all three mutant mice strains (TRPA1^-/-^, TRPV1^-/-^: p = 0.012, TRPV1/TRPA1 ^= / =^: p = 0.017, Fig 4B). BCTC (10 μM) effectively reduced CGRP release induced by 30 mM lidocaine in nerves of TRPA1^-/-^ mice (p = 0.002; Fig 4D). Again, HC030031 (100 μM) was not effective in preventing release of CGRP in nerves lacking TRPV1 (p = 0.401, Fig 4D). Similar to the data obtained with calcium imaging in DRG neurons, these data suggest that while 10 mM lidocaine more or less only employ TRPV1 and TRPA1 to evoke CGRP release, 30 mM lidocaine also employ mechanisms independent of TRPA1 and TRPV1 channels. Therefore, we next explored if lidocaine evokes an exocytosis of CGRP by releasing calcium from intra-axonal stores. Indeed, 30 mM lidocaine induced about the same amount of CGRP release in experiments with calcium-free extracellular solution as in the presence of calcium. Moreover, pre-incubation of sciatic nerves with thapsigargin (20 μM, 5 min) resulted in about 50% reduction of the subsequent lidocaine-stimulated CGRP release in calcium-free conditions. In contrast, thapsigargin did not reduce the effect of 30 mM lidocaine in the presence of calcium (both p = 0.028, Fig 4E).

### Lidocaine-evoked neurotoxicity in DRG neurons does not require TRPA1 or TRPV1 nor is linked to apoptosis

**Fig 5:**
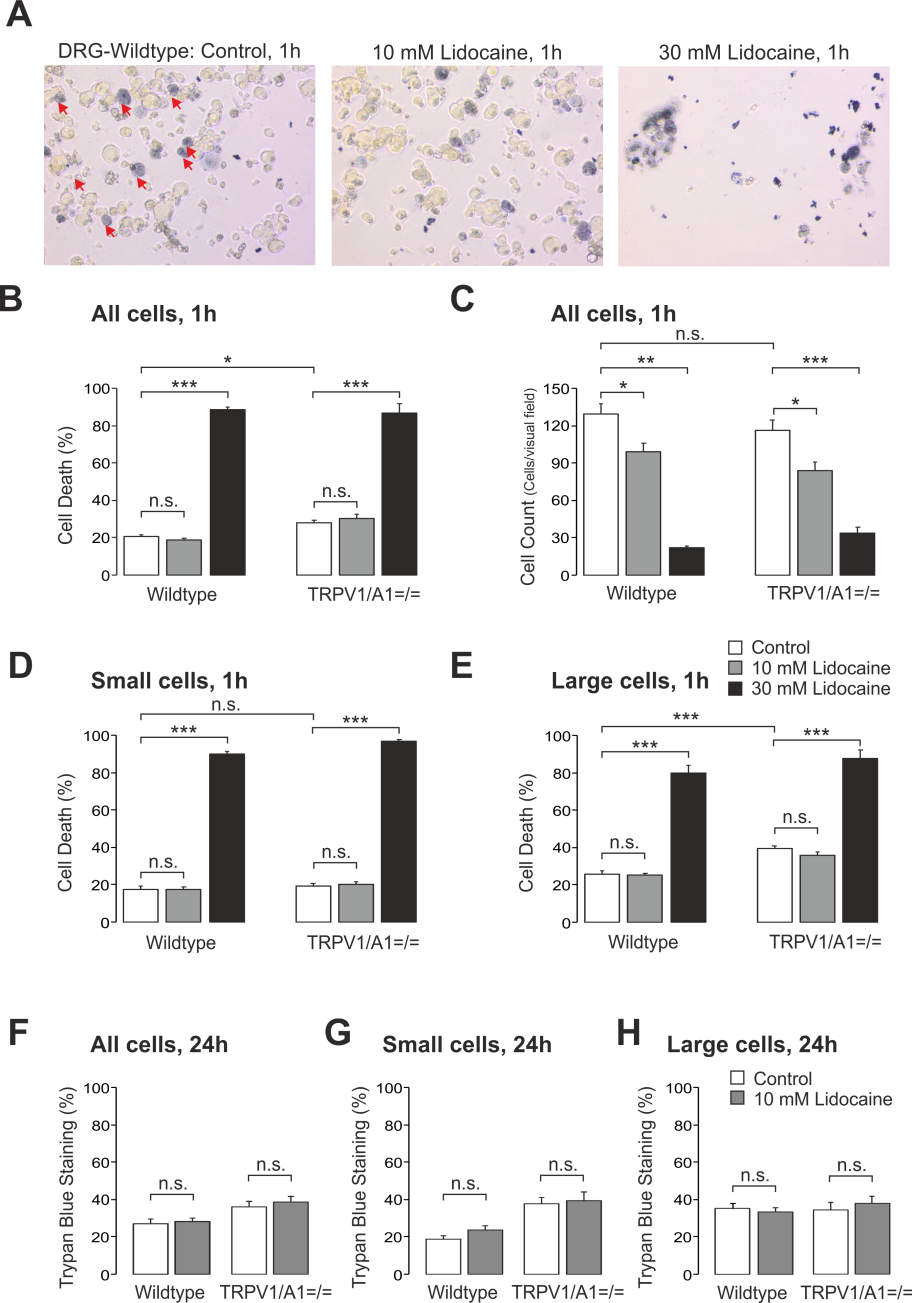
Lidocaine-evoked neurotoxicity does not involve TRPA1 or TRPV1 in DRG neurons. **A.** Representative phase contrast photographs (20x) of cultured DRG neurons stained with trypan blue (0.04%) following treatment with control solution (left, red arrows indicate cells stained with trypan blue), 10 mM lidocaine (middle) or 30 mM lidocaine (right). **B, D**-**H.** Bar columns displaying the mean percentage of dead DRG neurons from wild type or TRPV1/TRPA1 ^= / =^ mice (i.e. cells stained with trypan blue) before and 1 h or 24 h after treatment with 10 or 30 mM lidocaine. The cytotoxic effect of lidocaine was compared between the two genotypes in respect to all (B, F), to small (<30 μm, D, G) and to large (>30 μm, E, H) sized DRG neurons. **C.** Bar columns displaying the mean numbers of DRG neurons (counts/visual field) from wild type or TRPV1/TRPA1 ^= / =^ mice following treatment with 10 and 30 mM lidocaine. Data are presented as mean ± S.E.M. * indicates p< 0.001, *** indicates p< 0.001, n.s. not significant due to calculation with ANOVA followed by HSD post-hoc test.

Our findings so far indicated that TRPA1 and TRPV1 are relevant for lidocaine-evoked calcium influx in sensory neurons. It has previously been suggested that lidocaine-induced neurotoxicity on DRG neurons is partly mediated by calcium influx [9]. Therefore, it was next examined whether TRPA1 and TRPV1 are involved in lidocaine-induced cell death. To address this question, effects of 10 and 30 mM lidocaine on DRG neurons from wild type C57BL/6 and TRPV1/TRPA1 double knockout mice were analysed. Lidocaine was applied to the cell culture medium for 15 min and cell death was assessed by trypan blue staining 1 h or 24 h after lidocaine-treatment (Fig 5A). 10 mM lidocaine failed to increase cell death in wild type neurons (control: 20 ± 1%, lidocaine: 19 ± 1%, p = 0.98) and from mice lacking both TRPV1 and TRPA1 (control: 28 ± 1%, lidocaine: 30 ± 1, p = 0.93; Fig 5B). In contrast, 30 mM lidocaine induced nearly a complete cell death in DRG neurons of both genotypes (each p<0.001 versus control), thus, the absence of TRPA1 and TRPV1 did not reduce the neurotoxicity of 30 mM lidocaine (wild type: 89 ± 1%, TRPV1/TRPA1 ^= / =^: 87 ± 5%, p = 0.89, Fig 5B). Gold and colleagues have reported a concentration-dependent loss of DRG neurons from cover slips used for cultivation induced by lidocaine. The authors hypothesized that effects were probably due to detachment of dead neurons from the coverslips [9]. Accordingly, we observed that 10 mM lidocaine slightly and 30 mM profoundly reduced the numbers of cells from both wild type (control: 129 ± 9 cells, 10 mM lidocaine: 99 ± 7 cells, p<0.05, 30 mM lidocaine: 22 ± 2 cells, p<0.01) and TRPV1/TRPA1 ^= / =^ mice (control: 117 ± 8 cells, 10 mM lidocaine: 84 ± 6 cells, p<0.05, 30 mM lidocaine: 33 ± 5 cells, p<0.001, Fig 5C). Thus again, this neurotoxic lidocaine effect cannot be attributed to TRPA1 and TRPV1.

As TRPA1 and TRPV1 appear to be predominantly expressed in small to medium sized DRG neurons [13], cell death was separately evaluated in small (i.e. < 30 μm) and large (> 30 μm) DRG cells. However, even with this approach a role of TRPA1 and TRPV1 in cell death of small as well as large neurons induced by 10 or 30 mM lidocaine could not be detected (Fig 5D and 5E). Interestingly, untreated DRG cells exposed to only control solution showed an increased cell death if lacking both TRPV1 and TRPA1 (28 ± 1% dead cells) compared to wild type neurons (20 ± 1% dead cells, p<0.05, Fig 5B). This difference did not apply to small sized (n.s., Fig 5D) but to large sized neurons (wild type: 26 ± 5%, n = 24, TRPV1/TRPA1 ^= / =^: 39 ± 2%, n = 57, p<0.001, all ANOVA with HSD post-hoc test, Fig 5E). It remains unclear whether this result is of any relevance, i.e. if expression of TRPA1 and/or TRPV1 per se impairs cell viability in culture.

**Fig 6:**
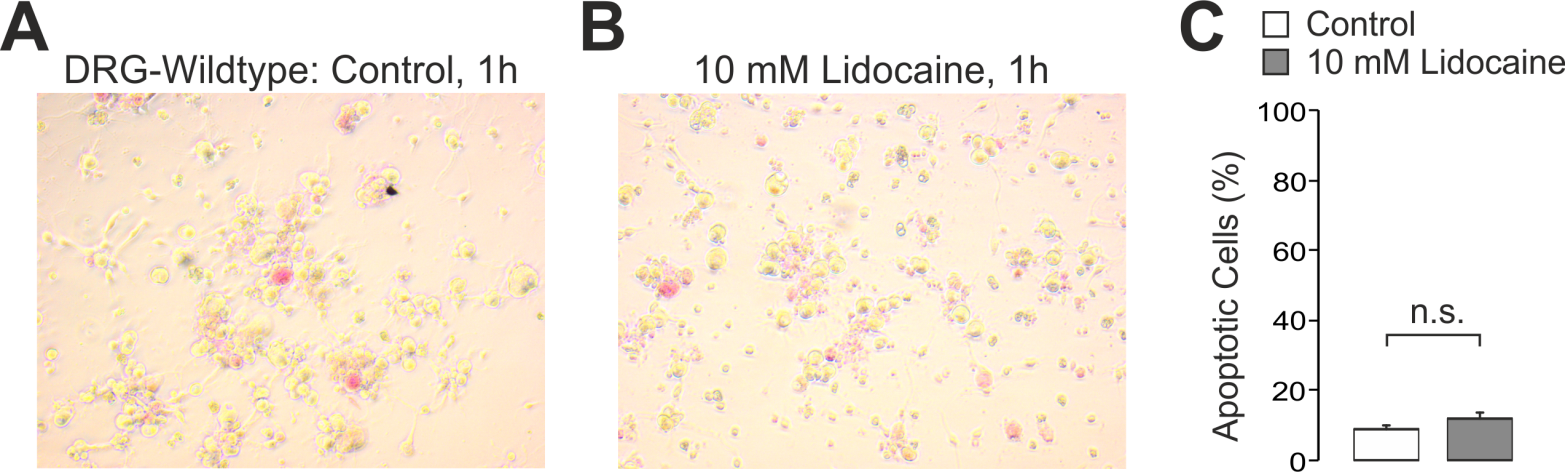
Lidocaine-induced activation of TRPA1 and TRPV1 does not result in apoptosis. **A** and **B.** Representative phase contrast photographs (20x) of cultured DRG neurons stained with the Cell-APOPercentage apoptosis assay subsequent to control (A) or 10 mM lidocaine treatment (B) for 1 h. **C.** Bar columns depicting the mean numbers of apoptotic DRG neurons (counts/visual field) following application of 10 mM lidocaine. Data are presented as mean ± S.E.M. n.s. was considered not significant by unpaired t-test.

Considering that a delayed onset of cell death due to apoptosis might not be observed within 1 h after incubation with lidocaine, experiments were also performed on DRG neurons which were allowed to recover for 24 h after treatment with lidocaine. As the very low number of cells remaining after 30 mM lidocaine hardly allowed any further interpretations, these experiments were limited to 10 mM lidocaine evoking a calcium-influx mediated only by TRPA1 and TRPV1. As is demonstrated in Fig 5F (n ≥ 16 coverslips, p = 0.72 resp. 0.99), G (n ≥ 10 coverslips, p = 0.99 resp. 0.72), and 5H (n ≥ 10 coverslips, p = 0.71 resp. 0.97), however, this approach also failed to induce a significantly increased trypan blue staining irrespective of genotype and cell size (all ANOVA with HSD post-hoc test). While staining with trypan blue identifies cell death, it has been reported that lidocaine-induced cytotoxicity in DRG neurons is predominantly due to apoptosis [22]. On this account we stained DRG neurons treated with 10 mM lidocaine for 1 h with the Cell-APOPercentage apoptosis assay using a dye that is selectively imported by cells undergoing apoptosis. As is demonstrated in Fig 6, apoptotic cells were determined in untreated cells (9 ± 1%, Fig 6A) as well as in cells treated with 10 mM lidocaine (12 ± 2%, Fig 6B). However, treatment with lidocaine did not significantly increase the number of apoptotic cells (n > 20 coverslips for each group, p = 0.15, unpaired t-test, Fig 6C).

Taken together, we found no evidence for a relevant involvement of TRPA1 and TRPV1 in lidocaine-evoked cell death and apoptosis in DRG neurons.

**Fig 7:**
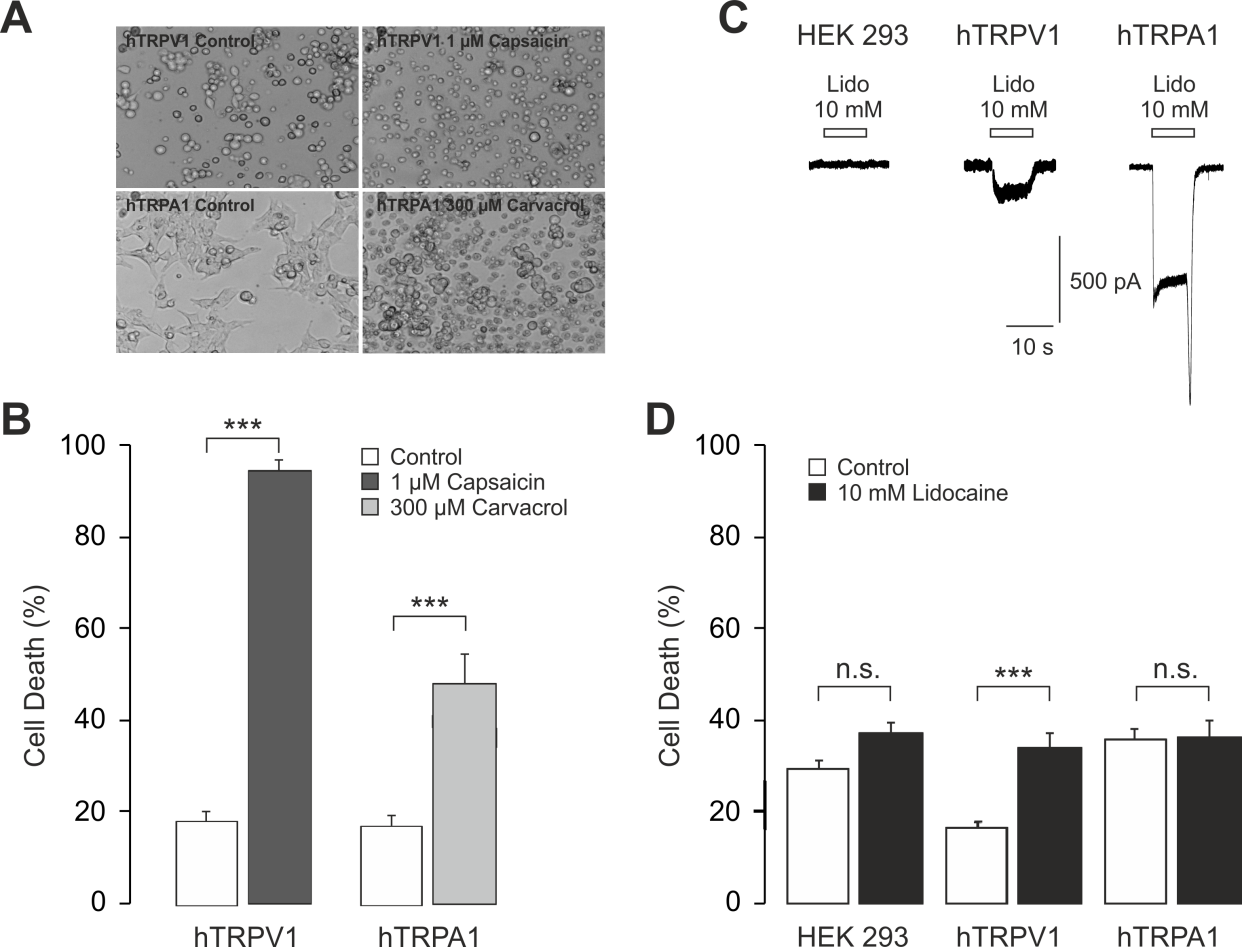
TRPV1, but not TRPA1 mediates lidocaine-induced cell death when overexpressed in HEK 293 cells. **A.** Representative phase contrast photographs (20x) of cultured HEK 293 expressing either hTRPV1 (upper row) or hTRPA1 (lower row) before (left) or after (right) treatment with 1 μM capsaicin or 300 μM carvacrol respectively. **B.** The bar columns display the mean percentage of dead cells (i.e. cells stained with trypan blue) before and after treatment with 1 μM capsaicin (hTRPV1) or 300 μM carvacrol (hTRPA1). Note that both agonists induce a significant cytotoxicity. **C.** Representative whole cell patch recordings displaying inward currents induced by 10 mM lidocaine in HEK 293 cells expressing hTRPV1 and hTRPA1, but not in untransfected HEK 293 cells. Cells were held at a holding potential of -60 mV. **D.** Bar columns displaying the mean percentage of dead untransfected HEK 293 cells, hTRPV1-expressing cells or hTRPA1-expressing cells following treatment with 10 mM lidocaine for 1h. Data are presented as mean ± S.E.M. *** indicates p< 0.001, n.s. not significant. Statistical comparisons were performed by ANOVA with HSD post-hoc test.

Several previous studies reported that both TRPA1 and TRPV1 can mediate cell death when activated by different agonists [15–18]. As TRPA1 and TRPV1 are expressed in a confined subpopulation comprising about 30–60% of the total number of mouse DRG neurons, it is possible that our approach to explore lidocaine-evoked cell death lacked sufficient sensitivity to detect a minor role of TRPA1 and TRPV1. Aiming to rule out the possibility that lidocaine-induced activation of TRPV1 and/or TRPA1 triggers cell death, we finally employed HEK 293 cells stably overexpressing hTRPA1 or hTRPV1. Treatment of hTRPV1-expressing HEK 293 cells with the TRPV1 agonist capsaicin (1 μM) for 1 h resulted in a strong cytotoxicity determined by trypan blue staining (control: 18 ± 3%, capsaicin: 93 ± 2%, p<0.001, Fig 7A and 7B). In HEK 293 cells expressing hTRPA1, 1 h treatment with the TRPA1 agonist carvacrol (300 μM) resulted in a significant cell death as well (control: 17 ± 2%, carvacrol: 48 ± 6%, p<0.001, both unpaired t-test). Carvacrol was used as TRPA1 agonist here since both AITC and acrolein induce a prominent cell death by TRP-channel independent mechanisms. As demonstrated in Fig 7C, application of 10 mM lidocaine on non-transfected HEK 293 did not evoke any inward currents. However, expression of hTRPV1 or hTRPA1 resulted in large lidocaine-evoked inward currents. When treating cells with 10 mM lidocaine for 1 h, it failed to induce a significant cytotoxicity in both non-transfected (control: 29 ± 2%, lidocaine: 37 ± 2%, p = 0.13) and hTRPA1-expressing HEK 293 cells compared to control (control: 36 ± 2%, lidocaine: 36 ± 4%, p = 1.0, Fig 7D). In contrast, cells expressing hTRPV1 displayed a significantly increased cell death following treatment with 10 mM lidocaine (control: 16 ± 2%, lidocaine: 34 ± 3%, p<0.001, all ANOVA with HSD post-hoc test, Fig 7D). Following treatment with 30 mM lidocaine of cells expressing hTRPA1, hTRPV1 or no TRP channels, a virtually complete cell death (i.e. 100%) was observed in all types of cells (data not shown).

## Discussion

In the present study we employed cellular assays to examine sensory neurons and sciatic nerves from mutant mice in order to unravel the roles of the irritant receptors TRPA1 and TRPV1 in lidocaine-evoked calcium influx, inward ion currents, release of neuropeptides and neurotoxicity. When administered for spinal or regional anesthesia at 1–2% (35–70 mM) in clinical practice, lidocaine is indeed applied at similarly high concentrations as those used in our study.

We found that both TRPA1 and TRPV1 significantly contribute to calcium influx and inward currents evoked by lidocaine, but that TRPA1 appears to be the most prominent mechanism in sensory neurons of mice. Independent of both TRPA1 and TRPV1, however, high concentrations of lidocaine also evoke a release of calcium from intracellular stores including the ER. The obvious role of TRPA1 and TRPV1 as the main molecular mechanisms for lidocaine-evoked influx of cations in sensory neurons probably has relevant implications. First, the local injection of lidocaine into the skin or mucous membranes is associated with a stinging and burning pain [11]. Although not a severe side effect limiting the use of lidocaine, it leads to a significant discomfort in patients. Our data imply that TRPA1 and TRPV1 are the main mechanisms for this painful sensation, as their natural agonists like the compound of mustard oil AITC and capsaicin induce pain of a similar quality. When applied intrathecally for spinal anesthesia, lidocaine most probably inhibits neuronal and, thus, synaptic activity primarily by blocking voltage-gated Na^+^ channels [1]. However, activation of both TRPV1 and TRPA1 in pre-synaptic central sensory nerve terminals might also result in a pre-synaptic inhibition due to a depolarization block of Na^+^ and Ca^++^ channels [23,24]. In agreement with our data suggesting that TRPA1 seems to be the most sensitive and, thus, most prevalent receptor for lidocaine, it was demonstrated that TRPA1 mediates lidocaine-evoked release of L-glutamate in central nerve terminals [25]. Thus, activation of spinal TRPV1 and TRPA1 channels might have both excitatory and inhibitory effects. The notion that activation of TRPA1 and TRPV1 by lidocaine can result in excitatory effects is further substantiated by our findings showing that lidocaine-evoked release of the neuropeptide CGRP is crucially mediated by both TRPA1 and TRPV1. In the periphery, the release of CGRP is associated with substance P release, and together these neuropeptides are responsible for ´neurogenic inflammation´, i.e. vasodilatation and plasmaextravasation which, however, do not sensitize sensory nerve endings [26]. Both neuropeptides are also known to contribute to central sensitization by acting on central CGRP and NK1 receptors in spinal neurons and glia cells of the dorsal horn [27]. When considering the widespread and unproblematic clinical use of lidocaine, however, we can hardly identify any obvious side effects of lidocaine which apply to a release of CGRP or substance P. Even more, rather than vasodilatation topical lidocaine (2%) causes a massive decrease of peripheral nerve blood flow [28]. Spinal anesthesia with lidocaine at high concentrations (5%) was reported to be associated with, at least, transient neurologic symptoms including radiating pain [5]. While such side effects are commonly regarded to result from direct neurotoxic effects of lidocaine, it is possible that spinal neuropeptides might contribute to some of these neurological symptoms. This notion remains speculative, as lidocaine is in fact rather considered to induce relevant anti-inflammatory and even anti-hyperalgesic effects [29,30]. When applied onto peripheral sensory axons, 30 mM lidocaine was demonstrated to induce a long lasting desensitization of axonal TRPA1 channels [31]. Accordingly, our data from calcium imaging clearly show that lidocaine can induce a concentration-dependent desensitization of TRPA1 and TRPV1. Thus, lidocaine-evoked activation followed by desensitization of TRPA1 and TRPV1 might better match the clinical observations.

The probably most relevant side effect of lidocaine examined in this study is the neurotoxicity. Following early clinical observations reporting that high concentrations of lidocaine can induce reversible as well irreversible neurological symptoms [5,32–34], a large number of studies have been performed investigating the mechanisms mediating lidocaine neurotoxicity [7]. Lidocaine-evoked cell death seems to involve several intracellular signaling pathways including activation of caspase, phosphoinositide 3-kinase and mitogen-activated protein kinase [7]. However, little is known about the upstream mechanism(s) triggering this intracellular signaling leading to cell death. A possible key mechanism for lidocaine-evoked cytotoxicity is an initial increase in intracellular calcium, and it has previously been demonstrated that removal or buffering of extracellular calcium strongly attenuates lidocaine-induced cell death of rat DRG neurons [9]. Strong and/or prolonged activation of TRPV1 is associated with a calcium-dependent cytotoxicity [16], an effect which is employed to treat focal neuropathies with topical capsaicin plasters [15]. Interestingly, it could also be demonstrated that local treatment with lidocaine plasters is associated with a loss of intraepidermal nerve fibers [35]. Although little is known about the ability of TRPA1 to induce cell death, recent reports suggest that it also mediates cytotoxicity in oligodendrocytes, at least [17,18]. Our data propose that even if TRPA1 and TRPV1 are responsible for lidocaine-evoked calcium influx through the cell membrane in sensory neurons, they do not seem to mediate lidocaine-induced neurotoxicity. Thus, although 10 mM lidocaine evoked a robust TRP channel-dependent calcium influx, an increased cell death was not observed. At the higher concentration of 30 mM, however, lidocaine evoked a massive cell death which was independent of TRPA1 and TRPV1. This conclusion was substantiated by our experiments on neurons from mice lacking both TRPA1 and TRPV1, but also on wild type neurons showing nearly 100% cell death although less than 60% express both or one of the TRP receptors. This negative finding seems surprising and we cannot rule out that an *in vitro* approach on cultured DRG neurons is inappropriate for detecting a minor role of TRPA1 or TRPV1 in lidocaine-evoked neurotoxicity. However, we also determined that TRPA1 even failed to mediate lidocaine-indued cell death in HEK 293 cells with a strong overexpression of TRPA1. In contrast, overexpression of TRPV1 resulted in a small but significant increase in lidocaine-evoked cell death. Accordingly, we recently demonstrated that TRPV1, but not TRPA1, mediates cytotoxicity when the channels are activated by the lidocaine-derivative QX-314 [36]. These findings raise doubts as to whether TRPA1 can mediate cell death at all. Again, the involvement of TRPA1 in processes resulting in cell death may not be adequately studied in an *in vitro* cellular model. Two recently published studies suggesting a role of TRPA1 in hypoxia-induced myelin damage did in fact not show that cell death directly resulted from TRPA1 channel activation [17,18]. With TRPV1, however, there is little doubt that cell death is a direct result of channel activation leading to an increase in intracellular calcium. At this point we cannot explain why we failed to detect a presumed role of TRPV1 in lidocaine-evoked cell death of nociceptive sensory neurons, unless a minor role was overridden by the higher impact of another mechanism. Lidocaine-evoked cell death has been demonstrated in several cell lines obviously lacking expression of TRPV1 [22,37]. Thus, it is clear that TRPV1 is not required for lidocaine-induced cell death. Interestingly, our data also show that 30 mM lidocaine is able to increase intracellular calcium by mobilizing intracellular stores. This effect was previously demonstrated in the neuronal cell line ND7/23 which lacks expression of TRPA1 and TRPV1 [10]. When depleting those intracellular calcium stores with thapsigargin, as was also possible in our experiments, the cytotoxicity of lidocaine even increased [10]. The authors also demonstrated that even higher concentrations of lidocaine (2.5–5% = 80–160 mM) evoked cell death by inducing a sustained calcium influx through the cell membrane. The mechanism mediating this calcium influx was obviously independent of TRPA1 and TRPV1, and it was suggested that it may be due to unspecific detergent-like properties of lidocaine resulting in membrane disruption [38]. Even though we did not specifically further address that notion in this study, our data at least do not offer a more plausible mechanism by which lidocaine at high concentrations may induce cell death.

In summary, our data demonstrate that both TRPA1 and TRPV1 expressed in peripheral sensory neurons are mandatory or partly responsible for calcium influx, membrane depolarization and release of neuropeptides upon application of clinically relevant concentrations of lidocaine. While our data do not support a relevant involvement of TRPA1 or TRPV1 in lidocaine-induced neurotoxicity, they indicate that further research on the possible role of primarily TRPV1 in lidocaine-evoked neurotoxicity is necessary before final conclusions can be drawn.

## Abbreviations

AITC: Allyl isothiocyanate
BCTC: 4-(3-chloro-2-pyridinyl)-N-[4-(1,1-dimethylethyl)phenyl]-1-piperazinecarboxamide
CGRP: Calcitonin gene-related peptide
DRG: Dorsal root ganglia
HEK: Human embryonic kidney
LA: Local anesthetic
SIF: Synthetic interstitial fluid
TRP: Transient Receptor Potential
